# Potential of Food Hydrolyzed Proteins and Peptides to Chelate Iron or Calcium and Enhance their Absorption

**DOI:** 10.3390/foods7100172

**Published:** 2018-10-19

**Authors:** Mallory E. Walters, Ramak Esfandi, Apollinaire Tsopmo

**Affiliations:** 1 Food Science and Nutrition Program, Department of Chemistry, Carleton University, 1125 Colonel By Drive, Ottawa, ON K1S 5B6, Canada; MalloryWalters@cmail.carleton.ca (M.E.W.); RamakEsfandi@cmail.carleton.ca (R.E.); 2 Institute of Biochemistry, Carleton Unive6rsity, 1125 Colonel By Drive, Ottawa, ON K1S 5B6, Canada

**Keywords:** protein hydrolysates, mineral binding, spectroscopic characterization, bioavailability, Fourier transform, ultraviolet

## Abstract

Iron and calcium are two essential micronutrients that have strong effects on nutrition and human health because of their involvement in several biological and redox processes. Iron is responsible for electron and oxygen transport, cell respiration, and gene expression, whereas calcium is responsible for intracellular metabolism, muscle contraction, cardiac function, and cell proliferation. The bioavailability of these nutrients in the body is dependent on enhancers and inhibitors, some of which are found in consumed foods. Hydrolyzed proteins and peptides from food proteins can bind these essential minerals in the body and facilitate their absorption and bioavailability. The binding is also important because excess free iron will increase oxidative stress and the risks of developing chronic diseases. This paper provides an overview of the function of calcium and iron, and strategies to enhance their absorption with an emphasis on hydrolyzed proteins and peptides from foods. It also discusses the relationship between the structure of peptides and their potential to act as transition metal ligands.

## 1. Introduction

Foods are composed of nutrients, non-nutrients, and phytochemicals. Amongst the nutrients, proteins have functional attributes that can facilitate processing and cooking, and enhance the performance of the food product. Major functional properties of food proteins include water and oil binding, emulsification capacity (i.e., surfactant), foam stability, and viscoelastic characteristics [1,2]. In humans, proteins mainly provide the building blocks of tissues upon digestion and absorption, but can also serve as a source of energy. Protein hydrolysates have been studied in the past decades and are considered an alternative to intact proteins in the formulation of foods to meet nutritional requirements of people in different stages of development or with different health statuses [3,4,5]. Hydrolyzed proteins and derived peptides also possess several biological activities, such as antioxidant, anti-inflammatory, anti-hypertensive, and mineral binding [6,7]. 

Metals are important in a wide variety of biological processes and their roles extend from nutritional to the control of key pathways that affect growth and development. The efficacy of minerals including calcium and iron, which are the topic of this paper, is affected by how much is present in the biological system (i.e., absorption and transport). Ion transporters participate in the maintenance of the required level in different cellular compartments [8]. The amount of bioavailable calcium and iron may be decreased in individuals with clinical conditions where the expression of transporters is down-regulated [9,10]. Meanwhile, dietary absorption is considered to be the major factor affecting their bioavailability. The presence in some food matrices of components such as phytates constitutes the major dietary impediment to the uptake of divalent metals (e.g., Ca^2+^, Fe^2+^, and Zn^2+^). This is due to the propensity of phytates to form insoluble complexes with the divalent metals rendering them unavailable, as there is a lack of phytate-hydrolyzing enzymes in the human gastrointestinal tract [11]. It is therefore challenging to recover free calcium or iron from such complexes and deficiency can occur. Although the concentration of phytates can be reduced by adding commercial phytase and leavening agents, fermentation, germination, and milling [12,13], not all foods can be processed in these ways. The consumption of foods containing large amounts of other minerals (e.g., zinc, copper) can also affect calcium and iron absorption due to their competition for common transporters and carrier proteins [14]. Deficiencies of these minerals can lead to diseases that affect many organs and functions. A low circulating amount of calcium, for example, leads to a release of calcium from bone and increases the risk of osteoporosis. Meanwhile, when iron levels are low, consequences include anaemia, impaired physical activity and endurance in adults, and cognitive impairment in children [15,16]. A seemingly adequate intake of dietary calcium and iron still poses some concern because an inadequate amount can be absorbed in the small intestine, due to precipitation and formation of insoluble salts in the neutral to the slightly basic intestinal lumen. To overcome this issue, metal salts and multi-mineral supplements have been used in the food industry, but there have been some adverse effects on food products. In some cases, fortification with ferrous sulfates, for example, might cause unacceptable colour changes in infant cereals and tortillas, or a metallic taste in fruit drinks or purees [17,18]. There are many polyphenols in fruits and plants, some which possess *ortho*-substituted hydroxyl groups that can form complex derivatives with iron and hence change the colour and taste of a product.

There has been greater interest in the potential of hydrolyzed proteins and peptides to bind calcium and iron, thereby enhancing their dissolution and subsequent absorption [19,20]. Hydrolyzed proteins and peptides are capable of binding divalent minerals and improving their stability, solubility, and bioavailability due to the presence of various amino acid residues with ionic and electron rich side chains, as well as proper conformations [21,22]. This binding property also enables hydrolyzed proteins and peptides to function in the prevention or termination of free radical-mediated oxidative reactions facilitated by the pro-oxidant nature of divalent metal ions, such as Fe^2+^ and Cu^2+^ [22,23]. The metal-chelating capacity is also relevant in human nutrition because they can facilitate the absorption of chelated calcium and iron in the digestive tract. Knowledge of the structural requirements for metal chelation by hydrolyzed proteins and peptides is important to facilitate their application as dietary mineral carriers. This review discusses the calcium- and iron-binding properties of hydrolyzed food proteins and peptides, structural requirements, and enhancing effects on absorption.

## 2. Fundamental Roles of Calcium and Iron

Calcium is the most abundant mineral in the human body, with a prominent function in the mineralization of bones and teeth, while iron is responsible for functions such as the transport and storage of oxygen, production of red blood cells, and catalytic cofactors of oxidoreductases [24,25]. Calcium is also required for physiological functions, such as oxidative phosphorylation, and the maintenance of a healthy body weight [26], blood clotting, muscle contraction, cell division and membrane functions, enzyme activity, nerve transmission, and hormone secretion [16,27]. Typically, calcium facilitates the regulatory functions through its binding ability to proteins, like to troponin C, to stimulate muscle contraction and in addition to osteoporosis, its deficiency is linked to hypertension and some forms of cancer [28,29]. 

A proper amount of iron is crucial for cell respiration, DNA/RNA synthesis, and gene expression, while its deficiency can lead to anemia, depression, and nervous and muscles developmental delay in children [30,31]. Free ferrous ions (Fe^2+^) react with hydrogen peroxide to produce highly reactive hydroxyl radicals through a process known as the Fenton reaction. The generated hydroxyl radicals tend to damage biomolecules such as proteins, lipids, and DNA/RNA [22]. A specific form of iron-dependent oxidative cell termed ferroptosis has been described in mammalian and fibroblast cells [32]. It can be triggered in biological systems by antitumor molecules such as erastin and sulfasalazine. Both molecules inhibit the uptake of cysteine and consequently the synthesis of the antioxidant peptide glutathione [32,33]. Cells then die from excessive lipid peroxidation due to the iron-dependent accumulation of reactive oxygen species. Although this mechanism is important in tumor cells, it is damaging to normal cells. Antioxidants such as vitamin E and its derivate, trolox, can prevent ferroptosis, but iron chelators such as deferoxamine and ciclopirox olamine are effective as well [32,34]. It will be interesting to test other chelators, such as food protein-derived peptides specifically, because some may also possess antioxidant activities.

## 3. Absorption and Bioavailability of Calcium and Iron

Calcium and iron are available in organic and inorganic forms, which influence their absorption and bioavailability. Calcium is absorbed in the duodenum via transcellular active transport when its concentration is low, and in the ileum and jejunum via passive transport when the concentration is high [35]. Casein phosphopeptides (CPPs) are derived from milk proteins and enhance calcium, iron, and zinc absorption. Vitamin D can enhance calcium absorption, as well through the activation of calbindin-D9k, a membrane-bound calcium transporter [36]. Dietary iron is available in the heme form found in animal products and the non-heme form present in plants. Heme releases iron upon proteolysis in the stomach, after which it is more easily absorbed by enterocytes. Non-heme iron is less absorbed due to the presence of antinutrients in plants, such as phytates, oxalates, and tannins [37]. It is believed that iron is either released from heme in the intestine upon digestion or that it is released intracellularly when intact heme is taken up by cells. Some studies have found heme-binding proteins within the brush border and intestinal enterocytes that act as transporters or facilitate transport [38]. Ferric iron Fe^3+^ is poorly soluble, and is thus less bioavailable than its reduced form, Fe^2+^. Meanwhile, vitamin C enhances iron absorption through the conversion of Fe^3+^ to Fe^2+^. 

Many dietary factors may inhibit or enhance the absorption and the bioavailability of calcium, iron, and other divalent metal ions. Some examples of inhibitors are phytates, oxalates, and the excessive presence of other cations, such as zinc and magnesium. Phytates and oxalates are chelators and humans lack the enzyme phytase to release the trapped minerals [39]. Polyphenols, a known group of antioxidant molecules, can also form complexes with iron or calcium and inhibit their absorption [40]. The anti-nutritional effect of phytates can be reduced by fermentation, cooking, and baking, which reduce available binding sites of phytates [41]. Due to the physiological importance of iron and calcium, the development of other strategies to combat their low bioavailability is important. Ferrous sulfates, ferrous lactate, ferrous gluconate, and ferric ammonium citrate minerals have been added to foods, but undesirable sensory characteristics were detected in some products [18]. Individual amino acids such as cysteine and histidine are also capable of chelating or reducing ferric iron, but their excess can decrease the absorption of other amino acids. Because of this, the use of polypeptides as a metal chelator is a good alternative. 

## 4. Methods to Determine Binding and Absorption of Calcium and Iron

The binding or chelation of metals is an important step in the study of their absorption and bioavailability. Such actions can prevent precipitation in the lumen or protect them from inhibitors present in various foods. Methods to determine the chelation of iron and calcium are based on the competitive binding of hydrolyzed proteins or peptides with known complexing compounds, followed by detection by selective ion electrode, titration, or atomic absorption spectroscopy. Initial steps always involve the mixing of hydrolyzed peptides with the metal solution. The iron solution is generally prepared from FeCl_2_, FeSO_4_, or (NH_4_)_2_Fe(SO_4_)_2_ [42], while that of calcium is made from CaCl_2_ or CaCO_3_ [19,43]. 

Two complexing agents are used for iron. The first and most widely employed one is based on a colorimetric procedure developed in 1971 by Carter [44] to measure the chelation of iron in serum samples. The coloured complex, Fe^2+^-ferrozine had maximum absorption at 562 nm. The decrease in absorbance in the presence of peptides is used to determine the chelating activity. The other compound, 2,2’-bipyridine, works in the same way and the decreased colour of the red complex at 522 nm allows the estimation of the chelating activity [45]. Titration with ethylenediametetracetic acid (EDTA) is an old, yet effective, way of measuring the calcium- and iron-binding of molecules, including peptides. The addition of peptides to a calcium solution binds a certain amount, while the unbound calcium is then titrated with EDTA until the colour of the indicator (xylenol orange) changes from purple to yellow [46]. Cobalt (II) sulfate is also an adequate indicator and the colour will change from deep blue to light pink upon titration [47]. It is also possible to quantify the unbound calcium remaining in solution after the addition of peptide using a Ca^2+^ selective ion electrode [48]. Colorimetric methods using *ortho*-phenanthroline for iron [49] and *ortho*-cresolphthalein complexone reagent for calcium [43] can determine the content of the unbound minerals and hence, the binding capacity of the peptides. Alternatively, peptides bound and unbound to iron or calcium can be accurately determined using flame atomic absorption spectrophotometry. 

Human intestinal epithelial Caco-2 cells that differentiate to mimic the brush border lining of the duodenum are used as a cellular model to evaluate the absorption and bioavailability of calcium, iron, and other nutrients, while rats are often used for in vivo studies. During cell experiments, calcium/iron salts and their peptide chelates are added to the apical region. Contents of the basolateral region are collected after a certain time for transport studies, but cells are also harvested for determination of their calcium and iron contents using fluorescence spectroscopy or atomic absorption [50,51]. Absorption in animal models has been conducted in various tissues and fluids by atomic absorption spectroscopy [52].

## 5. Hydrolyzed Food Proteins and Peptides that Chelate or Enhance the Absorption of Calcium and Iron

The breakdown of proteins into small fragments is an efficient way to improve their functionality and to generate bioactive peptides. Proteases are the most common hydrolyzing agents, but microbial organisms are used as well. The properties of hydrolyzed proteins vary according to the type of protease used, but also with the pH, temperature, duration of hydrolysis, and the enzyme-substrate ratio [53,54]. In many cases, a combination of these conditions is used to produce hydrolyzed proteins and peptides with desired functionalities, such as antioxidant, anti-inflammation, and anti-hypertensive activities; modulation of the immune system; and the chelation of minerals [6,7]. Starting materials are typically of animal, marine, and plant origin. Some are industrial by-products, such as dairy whey, fish skins/bones, cereal brans, and animal blood, for the purpose of value addition and waste reduction [55]. The enhancing effect on the bioavailability of essential minerals such as calcium and iron is due to increased solubility and protection from inhibitors in the lumen [56]. 

### 5.1. Animal Sources

Caseins, major proteins in cow milk, are phosphorylated at clusters of serine residues in relatively close proximity in the primary structure. The treatment of caseins with trypsin or chymotrypsin releases CPPs which are mostly known for their ability to bind calcium [57,58], but also iron and zinc [59]. CPPs, therefore, play an important role in the absorption and bioavailability of these minerals. CPPs possess different chelating capacities depending on whether they are released from α_s1_-, α_s2_-, or β-casein sub-units. For example, the uptake of iron complexed to CPPs from β-casein was higher than from an α_S1_-CPPs-iron complex [59]. The difference is likely due to the chemical structure of peptides, as the authors found that inhibition of the intestinal phosphatase significantly increased the uptake and the absorption of β-CPPs-iron, but had no effect on α_S1_-CPPs-iron. CPPs are commercially available for use in milk-based formula as dietary carriers of calcium and other divalent minerals due to the presence of a unique cluster of three phosphorylated serine residues and two glutamate residues [60]. Egg yolk also contains phosphorylated protein, like phosvitin, which was used to produce phosphopeptides with a binding capacity towards calcium and improve bioavailability [61]. 

Hydrolyzed proteins from many by-products of fish, mainly skins and bones, have shown metal chelating capacities, with gelatin and collagen proteins being the frequent sources of derived chelating peptides. The flesh and skeleton from a processed fish frame (*Johnius belengerii*) were used to obtain a peptide fraction through affinity chromatography separation. The extracted peptide solubilized a similar amount of calcium compared to commercial CPPs [62]. Under optimum conditions, sea cucumber ovum trypsin hydrolysates bind 53.5 mg of calcium per gram and form a complex made of compact nanoparticles that exhibit a folded and porous network structure [63]. In the case of iron, trypsin hydrolysate of gelatin extracted from the skin of farmed giant catfish chelated iron (9.93 mmol EDTA/g protein) better than the non-digested gelatin (5.43 mmol EDTA/g protein) [64]. The less than 3 kDa membrane filtered fraction of a commercial cod protein hydrolysate and its high performance liquid chromatography (HPLC) fractions chelated iron by up to 90% at 0.5 mg/mL [65]. Stonefish proteins hydrolysed under the optimum conditions (pH 6.5, 54 °C, enzyme/substrate ratio 1.5%, 360 min) chelated 25.1% of iron ions [66]. Other fish hydrolyzed proteins have calcium and iron chelating activities, as summarized elsewhere [55,67]. Like milk CPPs, many peptides in hydrolyzed proteins from fish skins and bones contain phosphate residues that can explain their good chelating capacity [68]. A variety of animal hydrolyzed proteins and derived peptides that do not contain phosphorylated moieties have calcium and iron chelating capacities. A list of some of them is provided in Table 1. 

### 5.2. Plant Sources

Cereals are an important source of energy in human nutrition and their processing generates a large amount of brans and germs that have been used to generate functional proteins and bioactive peptides. Proteins from rice brans treated with a combination of alcalase and flavourzyme achieved an iron chelating capacity of up to 83%, depending on the duration of hydrolysis [89]. Similar levels of iron chelation were obtained after the simulated gastrointestinal digestion of rice proteins [90]. Peptide FVDVT was recently identified as a strong calcium chelating peptide (89.9% at 5 mg/mL) in wheat germ hydrolyzed proteins after ultrafiltration, anion-exchange chromatography, gel filtration chromatography, and reversed-phase HPLC [80]. Other chelating hydrolysates from cereal by-products include oat brans [91] and barley [21]. Soy is an economically important crop that is often used to produce oil. The meal is rich in proteins that possess a very high digestibility and amino acid score amongst plants. These proteins are used as functional ingredients in many food preparations, but also as a source of bioactive peptides. Soy proteins hydrolyzed with protease from the bacterium *Chryseobacterium* sp. kr6 achieved an iron chelating capacity of 86% at 2 mg/mL, depending on the enzyme-substrate ratios [92]. Using validase FP and neutral proteases, Zhang et al. [93] found that fractions of hydrolyzed soy proteins exerted iron chelating activities in the ranges of 0.1–0.7 mg EDTA equiv./g, with fractions of molecular weight higher than 10 kDa being more powerful. A calcium-chelating peptide, DEGEQPFPFP, was isolated from soy protein hydrolysate in another work after affinity and reverse-phase separations [94]. Chelating capacities are expressed in different ways. It is difficult to compare percentages because different concentrations of hydrolysates, peptides, or reagents are often used. In addition, the distinction between stock and final concentrations is not always evident in many studies. A better comparison is possible when they are expressed as mg EDTA equivalent/g, or as mg of bound calcium or iron per gram of peptide.

### 5.3. Effect of Proteases on the Chelating Capacity of Hydrolysates

Various factors, including the amount and type of protease, and the duration of hydrolysis, affect the function of hydrolyzed proteins and peptides. The nature of the substrate (proteins) is equally important. In a study of Alaska pollock skin collagen, trypsin hydrolysates showed superior iron chelating activity compared to flavourzyme [82]. The authors explained the difference by the production of shorter peptides in the trypsin digests; unfortunately no information was provided for the size of peptides in the flavourzyme hydrolysates. Looking at their data, it is possible that the amount of enzyme 0.6% for trypsin versus 0.3% for flavourzyme was an important factor. A comparison of the calcium chelating properties of soy proteins hydrolyzed with four proteases found that the binding of two of the hydrolysates, protease M (66.9 mg Ca/g) and pepsin (60.6 mg Ca/g), was higher compared to neutrase (42.0 mg Ca/g) and flavourzyme (43.6 mg Ca/g) hydrolysates [19]. The authors found that the amount of bound calcium increased linearly with the carboxyl group content of hydrolysates and that deamidation with glutaminase further increased the binding. Barley proteins hydrolyzed with four proteases all bound calcium and iron ions and improved their solubility. Meanwhile, after 30 min digestion, the flavourzyme hydrolysate displayed the highest binding capacity for ferrous ions, while alcalase hydrolysate bound the most calcium relative to pepsin, trypsin, and flavourzyme digests [21]. 

The effect of the protease on the binding capacity is generally attributed to the size of peptides in the hydrolysates, which is often estimated using the degree of hydrolysis and gel electrophoresis. Although all studies recognized the importance of the sequences, it is almost impossible to elucidate the structure of all peptides in a hydrolysate, although few attempts have been achieved using peptidomics [22,52]. The effect of size has also been investigated by separating the hydrolysate using membranes, and depending on the protease, less than 1 kDa or more than 10 kDa can be the most active. As an example, the fractionation of flavourzyme hydrolysate improved the calcium chelating capacity from 46 μg/mL to 137 and 135 μg/mL for 1–5 and 5–10 kDa fractions, respectively, while it decreased the chelating capacity of the alcalase hydrolysate [21]. The importance of the sequence can be illustrated by the chelating capacity of similar selenocysteine tripeptides SAC (18.5 nmol Ca/µmol, 0.92 nmol Fe/µmol) and SCH (15.5 nmol Ca/µmol, 1.1 nmol Fe/µmol) from Alaska pollock skin collagen [69].

### 5.4. Structural Features that Affect the Chelating Capacity of Peptides

There are many functional groups in peptides that can contribute to their metal chelating capacity. The binding of calcium for example has often been reported to occur with phosphate and carboxyl groups [55], but these functional groups are also involved in the chelation of other divalent metals. Side-chain residues of amino acid residues within peptide structures such as cysteine, serine, histidine, aspartate, and glutamate, can serve as ligands for the binding of transition metals. The structures of these amino acid residues suggest that they might form a complex with peptides through electrostatic interactions or H-bond coordination. Many identified calcium and iron chelating peptides (Table 1) contain one or more of these amino acid residues; meanwhile, they displayed varying degrees of chelation due to their different structures (i.e., sequences). The *N*- and *C*-terminals of peptides can also participate in the binding interaction with metal ions [54]. The availability of amino acid residues for interaction with metals is pH-dependent, as is the net charge of a peptide, which may become neutral, negative, or positive, thereby hindering or promoting electrostatic interactions. Although milk CPPs are one of the best groups of metal-binding peptides, recent efforts have focused on identifying calcium- and iron-binding peptides from plants and animal hydrolyzed proteins. Techniques such as Fourier transform infrared (FT-IR), ultraviolet, and fluorescence spectroscopies, as well as nuclear magnetic resonance (NMR), have been used to study and characterize the structural motifs of calcium- and iron-binding peptides.

#### 5.4.1. Coordination of Peptides to Calcium and Iron Using FT-IR Spectroscopy

**Figure 1:**
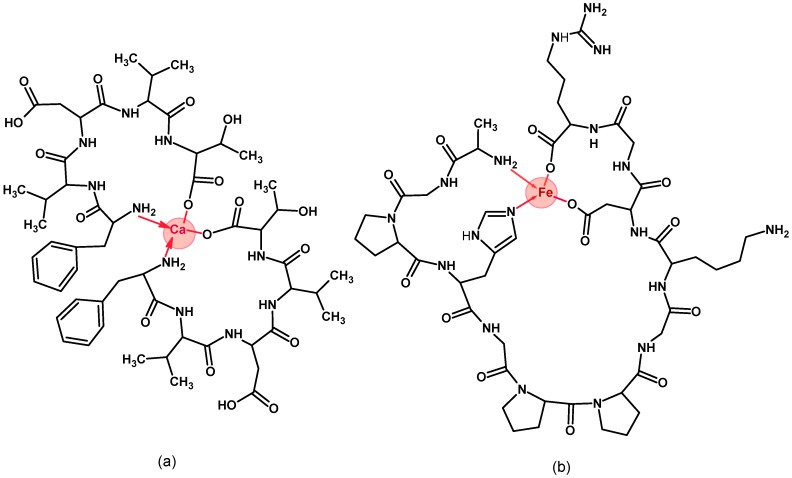
Possible calcium-binding structure for the peptide FVDVT (**a**) and iron-binding structure for the peptide AGPHGPPGKDGR (**b**).

The structure of polypeptides and their metal complexes are commonly investigated using FT-IR spectroscopy [95,96]. There are several useful functional groups in the IR spectra of polypeptides that can allow their characterization. Those referred to as amide I (1700–1600 cm^−1^), which consists of the stretching of C=O (peptide bond), coupled with amide II (60% N–H bending and 40% C–N stretching) near 1550 cm^−1^, and amide III (30% N–H bending and 40% C–N stretching) near 1300 cm^−1^, are widely used to study protein secondary structures [97]. FT-IR spectroscopy can also detect peptide side-chain moieties, such as aromatic rings, and carboxyl (-COOH), hydroxyl (-OH), sulfhydryl (-SH), and methyl (-CH_3_) groups, and potentially allows elucidation of the mechanisms underlying the binding of peptides to metals [96]. In a recent study, the mechanism of iron chelation by hydrolyzed proteins from sea cucumber was investigated using FT-IR. In the presence of iron, the amide N–H band shifted from 3303.2 to 3356.9 cm^−1^ and from 1649.7 to 1652.0 cm^−1^, which the authors attributed to the coordination of Fe^2+^ ions with electrons on the nitrogen atom and with the C=O group, respectively [88]. Although authors suggested the binding was primarily through interactions of iron with the carboxylate of aspartic acid, guanidine nitrogen of arginine, or nitrogen atoms in the imidazole group of histidine, this cannot be confirmed because it is not possible to determine the effect of the sequence when the chelating analysis is done on protein hydrolysates, as in this case. Similar interactions between amino nitrogen atoms and the oxygen of the carboxylate groups were used to justify the binding of Ca^2+^ ions by hydrolyzed tilapia fish proteins [98]. In the presence of calcium, vibrational stretching of N–H in peptide FVDVT shifted to a higher wavelength [80], suggesting a stronger electron cloud density around N–H due to the inductive effect or dipole field effect upon coordination. Other shifts detected for C=O and COO^−^ allowed the authors to suggest that amide and carboxylate groups were involved in the binding of calcium by FVDVT [80]. Similar shifts of N–H (stretching and bending) and C–N stretching bands to higher wavelengths, coupled with a decrease of C=O (peptide bond) and COO^−^ wavelengths, were used to explain the contribution of peptide bonds, and side-chain imine, terminal amino, and carboxylate groups to the iron chelating activities of three peptides (GPAGPHGPPGKDGR, AGPHGPPGKDGR, and AGPAGPAGAR) from fish skin gelatin [81]. Based on the data in these papers, possible structures of FVDVT calcium and AGPHGPPGKDGR iron complexes (Figure 1) show the coordination between amines (N-terminal) and carboxylates (C-terminal); meanwhile, the binding also occurs between amines and carboxylates on the side chain, the C=O of peptide bonds, and histidine. Using FT-IR spectroscopy, it is not possible to accurately determine chelating sites or the dominant ones. In addition, the imidazole moiety of histidine, which is important in the chelation of divalent metal ions, is not detectable using FT-IR. The limitation of FT-IR can be overcome by using Raman spectroscopy, which can provide considerable information to characterize the chemical environments of histidine and therefore its contribution as a ligand for polypeptide-metal interactions [99]. This is because Raman spectroscopy on histidine-containing polypeptides can differentiate between metal-coordinated and non-coordinated residues based on energy shifts in the vibrational modes of the imidazole moiety around 1570–1640 cm^−1^ [100]. The technique was used to show that histidine at certain positions favored *cis*-isomerization of human milk peptides and this was important for their radical scavenging activity, and demonstrated the involvement of histidine-rich peptides from mussels in the binding of nickel ions and their self-healing properties [101]. Meanwhile, no information was found on the use of Raman spectroscopy to study the mechanism of calcium- or iron-binding food peptides. 

#### 5.4.2. Coordination of Peptides to Calcium and Iron Using Ultraviolet or Fluorescent Spectroscopy

There are functional groups in hydrolyzed proteins and peptides that can provide structural information on their free forms and metal-chelates when assessed by UV or fluorescent spectroscopy. Carbonyl (C=O) groups that absorb at 190–230 nm in the UV region can be used to determine whether they are involved in the chelation of divalent metal ions. In a study by Zhao et al. [74], the shift of the band at 195 to 200 nm due to n→π transition of the carbonyl was used to explain the participation of the peptide bond in the chelation of calcium by the tripeptide YDT derived from whey proteins. Meanwhile, coordination of the peptide’s carbonyl bond of beta-lactalbumin hydrolysate to iron caused a shift from 204 to 196 nm [102]. The direction of the shift in UV absorption maximum then seems to depend on the nature of peptides. The majority of structural analyses have focused on the ability of aromatic amino acids to absorb or emit light. In the UV spectroscopy, peaks at 255–265 nm, 265–280, and 275–285 correspond to phenylalanine, tyrosine, and tryptophan residues, respectively [103]. There is an overlapping of tyrosine-tryptophan electronic interactions due to large bandwidths of UV absorption spectra, which limit structural characterization. To overcome the limitation, second derivative spectra, which highlight inflection points and shoulders of the absorbance spectrum of each of the three aromatic amino acids above, can allow the easier identification of peptides [104]. The derived spectra can also allow an assessment of the positions of these amino acids within the peptide sequence and their potential involvement in the chelation of metal ions. This technique has been used to determine the degree of hydrolysis and the homogeneity of tryptic casein hydrolysates with calcium-binding properties [105,106].

In the fluorescent spectroscopy of hydrolyzed proteins and peptides, phenylalanine, tyrosine, and tryptophan residues are natural chromophores and are responsible for their fluorescence [107]. Most works focus on tryptophan, which fluoresces at 310–340 nm, depending on the sequence, while phenylalanine (λ_max_ 280 nm) and tyrosine (λ_max_ 305 nm) are rarely used because of their relatively low quantum yield. The binding of metals to peptides containing tryptophan or tyrosine will cause a shift in maximum emission wavelengths or decrease peak intensities, which can be useful in the characterization of metal-peptide complexes. The addition of calcium to peptide YDT [74] or to cucumber seed hydrolyzed proteins [108] decreased fluorescence intensity and shifted emission wavelengths because of apparent folding that resulted in fluorescent residues being less exposed to the solvent. In related works, similar behavior was reported for iron-binding food peptides or hydrolysates, with time and pH having a significant impact on the changes [85]. Although fluorescent spectroscopy works have demonstrated that the binding of calcium and iron will result in the folding of peptides, available data cannot be used to fully characterize the metal-peptide complex.

#### 5.4.3. Coordination of Peptides to Calcium and Iron Using X-ray Diffraction or X-ray Absorption Spectroscopies

X-ray spectroscopy is a good method to study structural modifications to biologically active macromolecules such as proteins and polypeptides through changes in crystalline structures or redox states [109]. X-ray diffraction (XRD) spectra showed that the peptide FVDVT in its free state had a random amorphous structure characterized by three broad diffraction peaks [80]. In contrast, the calcium bound peptide had two sharp peaks, confirming crosslinking with Ca^2+^ ions. In another study, the XRD spectrum of a fraction of hydrolyzed fish proteins showed two diffraction peaks at approximately 7°–8° and 21°–23°. In the presence of calcium, the intensity of the first peak became sharp and narrow, illustrating crosslinking and an increase of the crystalline structure [98]. In the work of Hackett et al. [109], X-ray absorption spectroscopy (XAS) was used to determine the speciation of sulfur and alterations of its redox potential in brain tissues. These results show that XAS can be used to study the binding of sulfur-containing peptides to metals. The participation of sulfur will effectively result in changes in redox states and overcome the limitation of other methods that focus on carboxylates and aromatic ring moieties.

#### 5.4.4. Nuclear Magnetic Resonance

The distribution of electrons around proton, carbon, or phosphorous nuclei from NMR data can reveal the binding of metal to peptides. A proton NMR study of the complex formed between calcium and the dipeptide GY from a whey protein hydrolysate showed that signals of protons in the α-positions of the two amino acids shifted up field by 0.025 ppm [110]. This was an indication that the binding involved two molecules of the dipeptide and occurred with the N-terminal amine (-NH_2_) and C-terminal carboxylate (COO^−^), and not with the phenoxylate of tyrosine. The structure of the GY-calcium complex is relatively easy to draw because the dipeptide only possesses one amine and carboxylate, but with larger peptides, the difficulty increases. In a related work, although proton NMR revealed binding to amino and carboxyl groups of dipeptide FD, it could not distinguish whether it was with the aspartate (D) side chain or with the C-terminal [110]. When NMR experiments are performed, as in this case in deuterated water, there is an exchange of labile protons with deuterium and so protons of acid groups are absent from the spectra. Most works have then used NMR techniques to confirm the binding, rather than to determine the complete structure of the complex. For example, NMR data showed chemical shifts of the α- and NH-protons of the phosphoserine residues, but failed to show proton-proton correlations to fully elucidate the structure of the β-casein(1–25) calcium complex [111].

### 5.5. Enhance Absorption of Iron and Calcium by Hydrolyzed Proteins and Peptides

There is evidence that hydrolyzed proteins, fractions, and peptides of different sizes have an enhancing effect on the absorption and bioavailability of calcium and iron. The marine dipeptide PY from the marine plant *Schizochytrium* sp. enhanced the uptake of calcium by the intestinal Caco-2 cells by three-fold, while preventing the reduction of calcium absorption in the presence of known inhibitors such oxalate, phytate, and tannic acid, by 5.2-, 2.6-, and 2.7-fold, respectively [112]. The calcium was in a complex form with the peptide before the addition of the inhibitor and exposure to cells. Peptide GPAGPHGPPG from Alaska pollock fish improves iron uptake by 28% and calcium uptake by 113% in Caco-2 cells [50], while the peptide DKLPGFGDS*IEAQ (*phosphate) from egg white proteins showed a two-fold increase of iron uptake by Caco-2 cells and stimulated the iron-induced synthesis of ferritin [113]. A low molecular weight hydrolyzed meat protein fraction (<1 kda) enhanced iron uptake by Caco-2 cells, but not the larger molecular weight fractions, likely due to its stronger chelating capacity [114]. Peptide DHTKE from egg white improved calcium absorption across Caco-2 cell monolayers by more than seven times compared to the control CaCl_2_ [51], while SVNVPLY from hydrolyzed barley proteins increased the synthesis of ferritin and the uptake of iron by four-fold in Caco-2 cells [42].

In vivo data exist that show the positive effect of hydrolyzed proteins and peptides on calcium and iron absorption. The intragastric administration of the tilapia scale protein hydrolysate-calcium complex at the dose of 200 mg/kg per day significantly increased in a manner similar to CPP-calcium mineral density and the strength of femur bones compared to the control (CaCO_3_ only) during four weeks feeding [71]. In a related work, hydrolyzed collagen administered at 600 mg/kg to retinoic acid-induced bone loss rat models increased bone density, as well as the calcium content, by 22% (femur) and 12% (tibia), respectively, compared to those of the control rats [52]. Concerning iron, Lin et al. [83] found that chelating hydrolyzed proteins from hairtail fish species given to anemic rats had an effect and increased hemoglobin and ferritin concentrations; however, there was no difference with ferrous sulfate alone. The available data clearly show the enhancing effects of hydrolyzed proteins and peptides on calcium and iron in cellular and animal models. The actual mechanism is rarely investigated and therefore remains largely unknown. Some studies have found a relationship with the decrease of alkaline phosphatase [52,71], an enzyme that plays a major role in bone calcification, while elevated amounts are associated with liver or bone diseases. The uptake has been associated in some works with stimulation of the synthesis of the iron binding protein ferritin. Others have postulated that CPPs might interact with the plasma membrane and with or without the ion channel [115,116].

## 6. Conclusions

Research has shown a prospective solution to increase calcium and iron adsorption using hydrolyzed proteins and peptides. This is due to the ability of these food-derived molecules. Polypeptides possess many functional groups, which are important to their binding of divalent metals, as demonstrated by various spectroscopic analyses. Meanwhile, amongst similar functional groups, it is unclear which ones actually bind the metal. The increased absorption in cells and animals is clear, but more data is needed to fully elucidate the mechanisms. In humans, some works have shown the enhancing effect of CPPs on the bioavailability of calcium, but little attention has to been paid to other food protein-derived peptides. Further studies on the absorption of calcium, iron, and other minerals when they are chelated to peptides are of interest.

## Figures and Tables

**Table 1:** Hydrolyzed food proteins and peptides with calcium and iron chelating properties.

Bound Mineral	Source of Proteins	Enzymes Used	Sequenced of Tested Peptides	References
**Calcium**	Whey	Flavourzyme and protamex	GY	[43]
	Alaska pollock skin	Flavourzyme and trypsin	SAC, SCH, SGSTGH, and GPAGPHGPPG	[69]
	Pacific cod bone	Neutral and alkaline proteases (1:1)	None	[70]
	Tilapia fish	Pepsin, alcalase	None	[71,72]
	Wheat germ	Alcalase, protamex, flavourzyme, neutrase, and papain	None	[73]
	Whey proteins	Flavourzyme and protamex (2:1)	YDT, EG	[74,75]
	Antarctic krill	Trypsin	None	[76]
	Mung bean	Alcalase, flavourzyme, trypsin, pancreatin, and pepsin	LLLGI, AIVIL, PAIDL, and HADAD	[77]
	Algae	Alcalase and flavourzyme	FY, SSV	[78,79]
	Wheat germ	Alcalase	FVDVT	[80]
	Barley	Alcalase, flavourzyme, pepsin, and trypsin	None	[21]
**Iron**	Egg white	Alcalase	DHTKE	[51]
	Pacific cod skin	Trypsin	GPAGPHGPPGKDGR, AGPHGPPGKDGR,	[81]
	Fish skin	Flavourzyme, trypsin	None	[82]
	Alaska pollock skin	Trypsin	SAC, SCH, SGSTGH, and GPAGPHGPPG	[69]
	Barley	Selected and synthesis Synthesized (no enzyme)	SVNVPLY	[42]
	Hairtail fish proteins	Alcalase	None	[83]
	Walnut flake	Neutral protease	None	[84]
	Anchovy muscle	Flavourzyme, pepsin, papain, and alcalase	None	[85]
	Sugar-cane yeast extract	Alcalase, protex or viscozyme		[86]
	Mung bean	Alcalase, trypsin, pancreatin, and pepsin	PAIDL, LLGIL, AIVIL, LLLLG, HADAD	[77]
	Barley	Alcalase, flavourzyme, pepsin, and trypsin	None	[21]
	Whey	Alcalase, pancreatin, flavourzyme, and pepsin	None	[87]
	Sea cucumber	Alcalase, flavourzyme, papain, and trypsin	None	[88]
